# Carbon Material With Ordered Sub-Nanometer Hole Defects

**DOI:** 10.3389/fchem.2022.858154

**Published:** 2022-03-21

**Authors:** Nianjie Liang, Qiaosheng Li, Ganghuo Pan, Chunxiang Liu, Yuzhou Liu

**Affiliations:** ^1^ School of Chemistry, Beihang University, Beijing, China; ^2^ Beijing Advanced Innovation Center for Biomedical Engineering, Beihang University, Beijing, China; ^3^ Beijing Shenyun Zhihe Technology Co., Ltd., Beijing, China

**Keywords:** holey carbon material, hole defects, water-soluble, low bandgap, catalytic activity

## Abstract

A holey carbon material with ordered sub-nanometer hole defects was synthesized from oxidative cyclodehydrogenation of a polyhexaphenylbenzene precursor. Band gap of around 2.2 eV is formed due to the narrow connection between the hexabenzocoronene subunits. It has weak interlayer interaction energy compared with graphene and shows easy dispersion in a wide range of solvents, surprisingly including water. Density functional theory calculations confirmd the excellent dispersion of this material in water. This new carbon material was then proved as effective support for various inorganic nanoparticles of small sizes. The supported iron nanoparticles showed enzyme-like catalysis behavior in nitrophenyl reduction reaction by NaBH_4_, exemplifying the great potential of this new material in catalysis.

## Introduction

Graphene has garnered interest from people from all disciplines, because of its peculiar two-dimensional carbon-based conjugated structure (Geim and Novoselov, 2007). Graphene is a two-dimensional structure with sp^2^-honeycomb carbon lattice (Song et al., 2013). The formation of π bonds in the graphene honeycomb lattice creates a closed shell electron system. The superposition of multiple closed electronic shell systems produces an interaction dominated by repulsion, resulting in weak electronic coupling between the layers (Berashevich and Chakraborty, 2011). The weak interlayer interaction of graphene gives it a wide range of properties and applications (Lebedeva et al., 2011). The change of the force between graphene layers will have an important impact on its properties. Enhancing the interlayer interaction of bilayer graphene can effectively modulate the thermal transport (Sun et al., 2019). As the number of graphene layers increases, the interaction energy changes, and the room temperature thermal conductivity drops sharply (Ghosh et al., 2010). The interaction of bilayer graphene significantly changes the zero-field electronic structures, and has more abundant optical properties than monolayer graphene (Ho et al., 2010). Interlayer interaction will also affect the hydrogenation rate and selective etching (Imamura and Saiki, 2014). Exploring the interaction energy between graphene layers will help to prepare materials with novel functions.

The interlayer interaction of graphene is closely related to the dispersion of graphene. The energy barrier is introduced through electrostatic or steric repulsion to achieve electrostatically stability or steric stability and maintain a dispersion (Johnson et al., 2015; Hong et al., 2012). Many methods can be used to increase the dispersion of graphene. The graphite exfoliation (Yi et al., 2013; Bourlinos et al., 2009), microwave-assisted synthesis and functional modification methods can increase the dispersibility of graphene in water (Li et al., 2019; Long et al., 2011; Zuo et al., 2010; Wang et al., 2020; Jiang et al., 2019). By changing the solvent, graphite oxide can be stably dispersed in organic solvents (Mu et al., 2021), such as N,N-dimethylformamide (DMF) and tetrahydrofuran (THF). This can be explained as the polarity of the solvent molecules and graphene oxide sheets are comparable (Paredes et al., 2008). The selective adsorption of functional groups on graphene can increase its dispersibility in suitable solvents. The 1, 3-dipole cycloaddition can make graphene layers have higher dispersibility in water and DMF (Quintana et al., 2013). These methods affect the interlayer interaction of graphene, thereby improving the dispersion properties of graphene.

Graphene with sub-nanopores has a wide range of applications in ion transport (Suk and Aluru, 2014), selective ion sieving (van Deursen et al., 2019), seawater desalination (Xu et al., 2019), supercapacitors (Lee et al., 2016), etc., Graphene with nanopores has a strong water permeability and can be used as a reverse osmosis desalination membrane (Cohen-Tanugi and Grossman, 2014). The incorporate of three-dimensional nanopore crystals with sub-nano sized aperture size into the two-dimensional graphene laminate greatly improves the separation performance of water (Guan et al., 2017). Carbon materials with rich topological defects of high entropy have great potential in the field of electrocatalysis (Feng and Zhuang, 2020; Ding et al., 2021; Feng and Zhuang, 2021). Herein we would like to report our work of bulk synthesis of a graphene derivative with regular sub-nanometer defects. Correlation calculations confirm that its interlayer interaction energy is smaller than that of graphene, which leads to its unique dispersibility and electrocatalytic properties. Experiments and density functional theory (DFT) calculations confirmed that it can be dissolved in water well. Our work provides a new method for the dispersion and trapping of metal atoms in graphene in water.

## Preparation and Bandgap of Polyhexabenzocoronene Network

**FIGURE 1:**
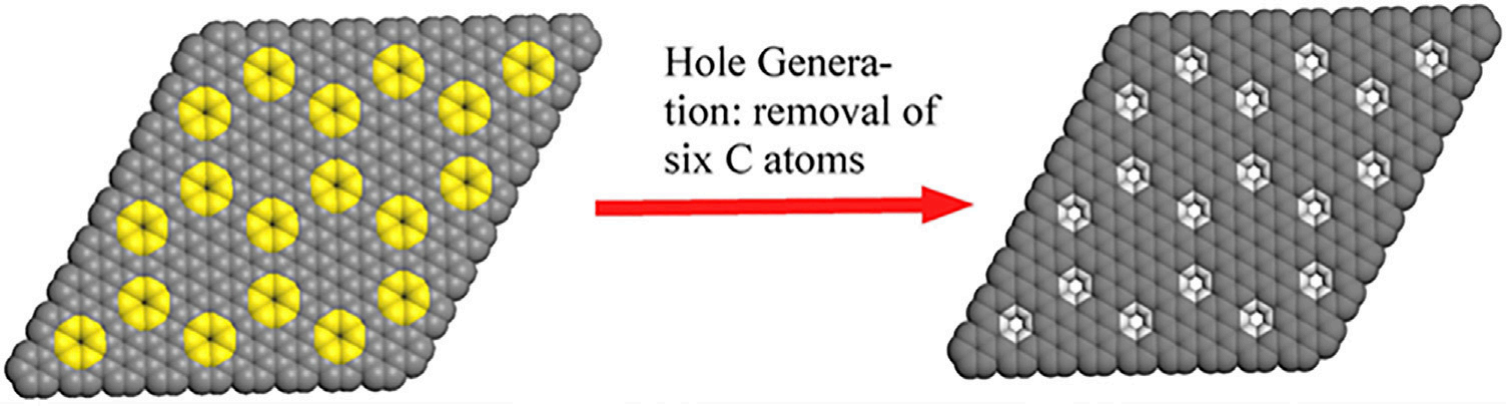
Design of holey graphene with sub-nanometer holes.

For bottom-up approach, the reaction condition in solution is much milder than that in previous top-down approaches, the formed small holes, which is found to self-heal under the high energy electron beam (Zan et al., 2012), are then expected to survive during the synthesis (Bieri et al., 2009). In addition, bottom-up approach offers structural precision and future tunability, since their structure follows deterministically from small-molecule precursors readily modified through organic synthesis. As shown in Figure 1, the removal of six adjacent carbons periodically from graphene layer will generate a holey graphene with sub-nanometer holes and hexabenzocoronene subunits. The hexabenzocoronene subunits are intentionally targeted due to the fact that relative hexaphenylbenzene precursors can be readily made. The careful design leads to extremely simple experimental procedure, and we surprisingly found that the designed holey graphene structure can be easily made in bulk through simple oxidation reaction of a topologically equivalent polyhexaphenylbenzene network (**PHN**). Upon be soaked in the CH_2_Cl_2_ solution of FeCl_3_ at room temperature, white **PHN** powder turned into dark brown immediately, which indicated the occurrence of the cyclodehydrogenation reaction. Finally, we obtained the anticipated polyhexabenzocoronene network (**PBN**). More synthetic details and structural confirmation are provided in our another manuscript (Liu et al., 2022).

**PBN** has a bandgap of around 2.2 eV, which matches very well with the calculated 2.1 eV at the HSE06 level and has a high accuracy in predicating bandgap energy. The bandgap is significantly reduced from those of hexabenzocoronene derivatives (2.7 ∼ 3 ev) (Hiszpanski et al., 2015; Hughes et al., 2012; Davy et al., 2016), again reflecting the highly conjugated nature of **PBN**. From the point view of holey graphene, the bandgap of this 2D hole-decorated graphene is justified by the narrow bridges between adjacent hexabenzocoronene units, whose width is inversely proportional to bandgap value (Son et al., 2006).

## Interlayer Interaction Energy of Polyhexabenzocoronene Network

**FIGURE 2:**
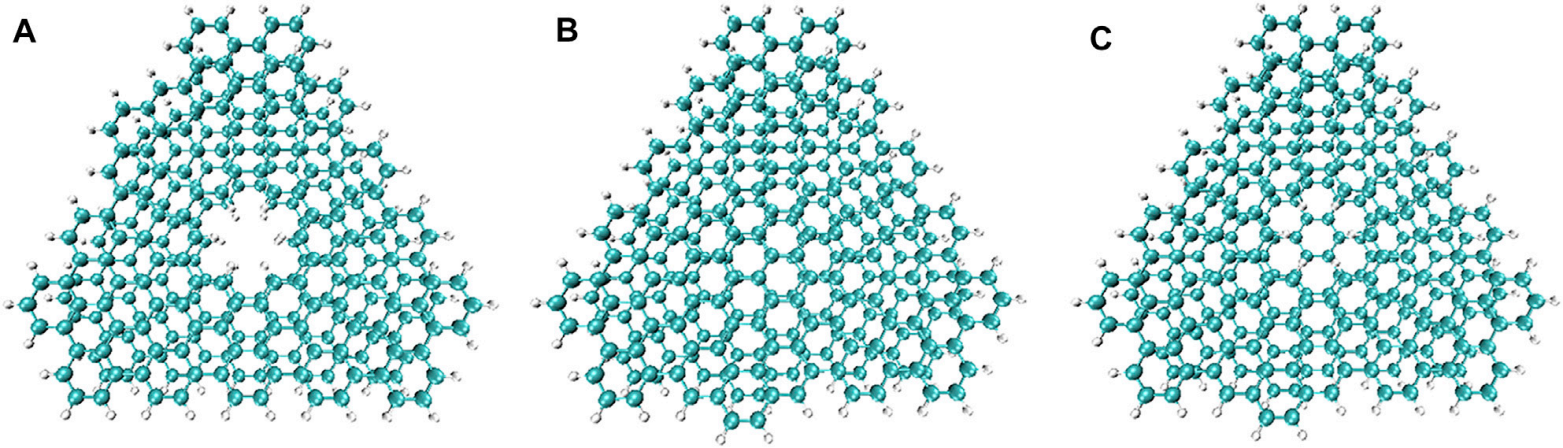
Structure diagram of **(A)** double-layer **PBN**, **(B)** double-layer graphene, and **(C)** one-layer **PBN** and one-layer graphene. The layer spacing is approximately 0.35 nm. Geometric structures were shown through VMD software (Humphrey et al., 1996).

Non-covalent interactions, including π-π interactions and van der Waals forces, are related to the dispersion of graphene (Georgakilas et al., 2016). Studying the non-covalent interaction between graphene and **PBN** can help explain the difference in the properties of the two. Independent gradient model (IGM) analysis, a method based on electron density (ED), can identify and isolate the interaction between user-defined fragments, using pro-molecular density (Lefebvre et al., 2017; Lu and Chen, 2022). Three models of double-layer **PBN**, double-layer graphene, one-layer **PBN** and one-layer graphene were considered to explore the difference of non-covalent interaction between **PBN** and graphene layers, as shown in Figure 2. The AA stacking structures were employed to qualitatively analyze the differences between the different models, ignoring the effect of different stacking models. The structure of the model was optimized first, and then the optimized structure was analyzed by IGM.

The structure optimization and single point energy calculation were performed with the ORCA package (Neese, 2012; Neese, 2018). The calculation used the BLYP functional, the def2-SVP basis set and the def2-SVP/J auxiliary basis set. The DFT-D3 dispersion correction with the Becke-Johnson damping function and tight SCF convergence criteria were used for calculation (Grimme et al., 2010; Grimme, 2011; Grimme et al., 2011; Becke and Johnson, 2006). The calculation solved the basis set superposition error (BSSE) problem by performing the geometrical Counterpoise Correction (gCP) at the level of a compound of the DFT and the def2-SVP basis set. The final single point energies after gCP correction of the three models and the individual layers of each model were counted. In order to describe the interaction energy between **PBN** or graphene, we define the interaction energy $\left(E_{\mathrm{interaction}}\right)$ as:

$$E_{\mathrm{interaction}}=\left(E_{\mathrm{total}}-\;E_{\mathrm{layer}1}-\;E_{\mathrm{layer}2}\right)/N_{\mathrm{atoms}}$$

where $E_{\mathrm{total}}$ represents the total energy of the three models, $E_{\mathrm{layer}1}$ and $E_{\mathrm{layer}2}$ represent the energy of **PBN** or **graphene** and $N_{\mathrm{atoms}}$ represents the total number of atoms of the three models.

**FIGURE 3:**
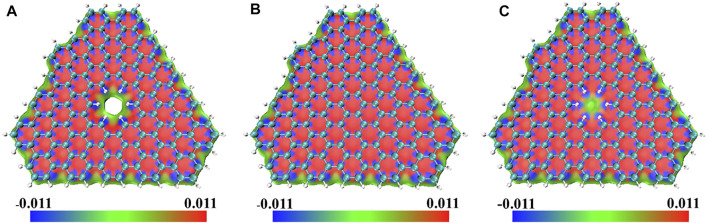
The sign $\left(\lambda_{2}\right)\rho$ colored map of $\delta g^{\mathrm{inter}}$ of **(A)** double-layer **PBN**, **(B)** double-layer graphene, and **(C)** one-layer **PBN** and one-layer graphene with isosurface of 0.008. Geometric structures were shown through VMD software (Humphrey et al., 1996).

It can be seen from Table 1 that the interlayer interaction energy of double-layer **PBN** is smaller than that of double-layer graphene, indicating that the existence of pores has an impact on non-covalent interaction. The interaction energy between one-layer **PBN** and one-layer graphene is slightly smaller than that of a double layer of graphene, indicating that the non-covalent interaction between the two pores is more obvious. In order to further explore the influence of pores on non-covalent interactions, IGM analyses were performed on the three optimized models using Multiwfn software 3.7 (Lu and Chen, 2012). In IGM analyses, δg^inter^ is used to describe the interaction between molecules, which is defined as:

$$\delta g^{\mathrm{inter}}=\left|\nabla \rho^{\mathrm{IGM,inter}}\right|-\left|\nabla \rho \right|$$

where |∇ρ| represents the norm of the ED gradient vector, and $\left|\nabla \rho^{\mathrm{IGM,inter}}\right|$ represents the upper limit of the independent gradient model when only the intermolercular interactions are cancelled. The sign of the Laplacian of the density, ${▽}^{2}\rho$ , can be decomposed into a sum of the contributions along the three principal axes of maximal variation. These components are the three eigenvalues $\lambda_{i}$ ( $\lambda_{1}$ , $\lambda_{2}$ and $\lambda_{3}$ ) of the ED Hessian (second derivative) matrix. The analysis of the sign of $\lambda_{2}$ can help discern different types of noncovalent interactions, and ρ provides the strength of noncovalent interactions, so the product of the two was used for complete analyses of noncovalent interactions (Johnson et al., 2010). Regarding the two layers of the three models in Figure 2 as two fragments for IGM analyses, the sign $\left(\lambda_{2}\right)\rho$ colored isosurface map of $\delta g^{\mathrm{inter}}$ was shown in Figure 3.

**TABLE 1:** Interaction energy statistics of different models.

Energy	Double-layer PBN	Double-layer graphene	One-layer PBN and one-layer graphene
$E_{\mathrm{total}}$ (au)	−11167.97	−11618.09	−11393.03
$E_{\mathrm{layer}1}$ (au)	−5588.86	−5808.91	−5808.91
$E_{\mathrm{layer}2}$ (au)	−5583.86	−5808.91	−5583.86
$E_{\mathrm{interaction}}$ (eV/atom)	−0.0179	−0.0192	−0.0188

In the blue area in Figure 3, the value of the sign $\left(\lambda_{2}\right)\rho$ was small and there was a strong attraction effect, where the value of the sign $\left(\lambda_{2}\right)\rho$ is close to 0, where there was van der Waals effect, and where the value of the sign $\left(\lambda_{2}\right)\rho$ was large, there was a strong mutual repulsion effect (Lu and Chen, 2021). In the vertical structure of the upper and lower layers, there was a strong attraction between the atom pairs, and there was a strong mutual repulsion in the hexahedral structure surrounded by carbon atoms. Where H atoms existed, such as holes and the edges of the system, there were Van der Waals forces. The existence of pores caused the non-covalent interaction to change from strong repulsion to van der Waals interaction, which reduced the interlayer interaction energy. This effect was only obvious in the vicinity of the pores. The weak interlayer interaction energy leads to some unique properties of **PBN**.

## Dispersion of Polyhexabenzocoronene Network

**FIGURE 4:**
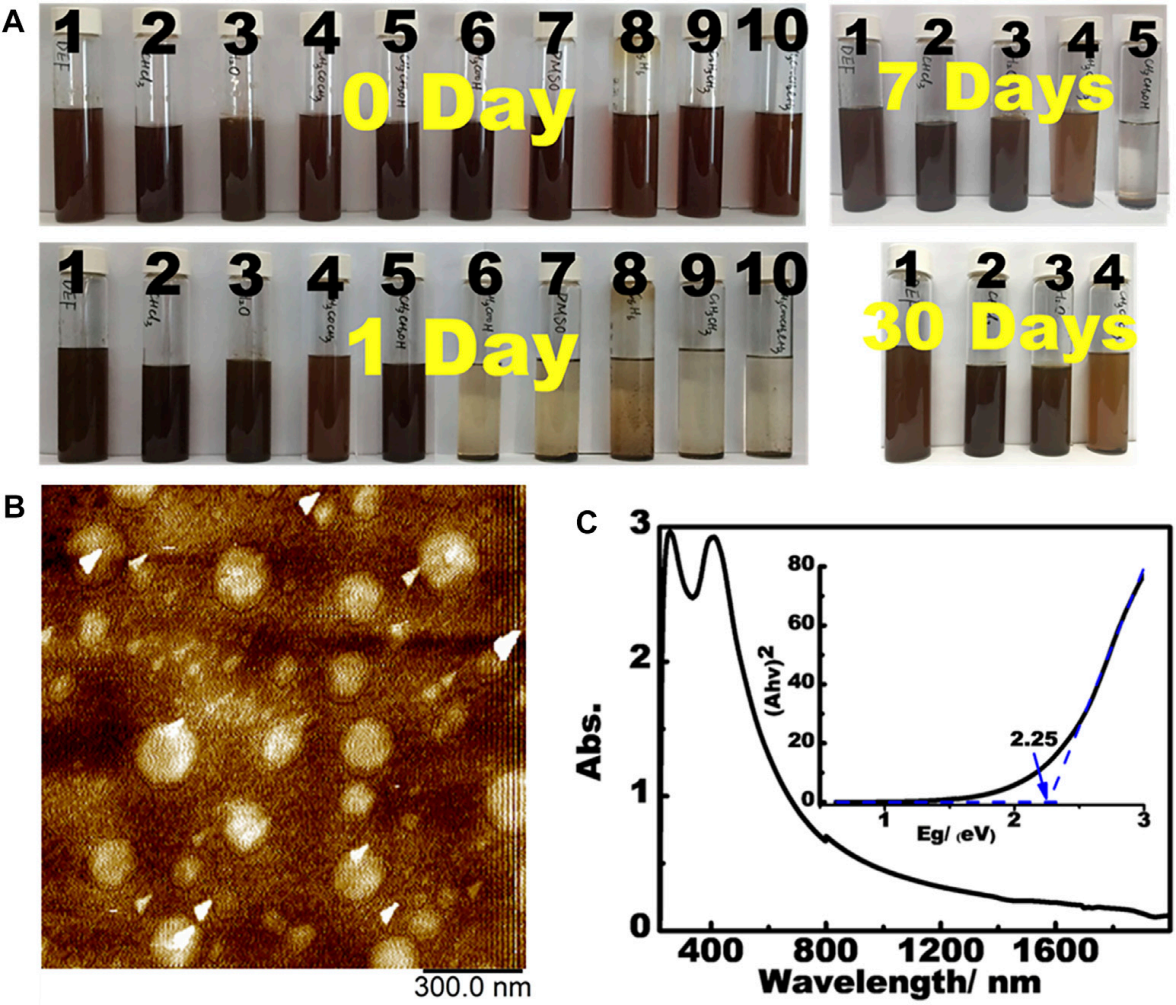
**(A)** The dispersion of **PBN** in various solvents (10 mg/20 ml). Solvent list: **(1)** diethylformamide, **(2)** chloroform, **(3)** water, **(4)** acetone, **(5)** ethanol, **(6)** acetic acid, **(7)** DMSO, **(8)** cyclohexane, **(9)** toluene, and **(10)** ethyl acetate. **(B)** AFM images of aqueous dispersion. **(C)** UV-Vis-Near IR spectra of **PBN** with the insert showing the band gap calculation.

**PBN** can be readily dispersed in a wide range of solvents. Unlike graphene and most 2D materials, **PBN** spontaneously disperses in common organic solvents to form dark red dispersion upon sonication (Figure 4A). The stability of the dispersion is also adjustable by the choices of solvents, with the dispersant ability roughly following the order of DEF, CHCl_3_, H_2_O > Acetone > Ethanol > HOAc, DMSO, cyclohexane, toluene, EtOAc. AFM analysis on the aqueous dispersion show plates with the heights of 1∼2 nm and diameters of up to 200 nms (Figure 4B), reflecting the few-layer dispersion in water. As far as we know, **PBN** represents the first 2D material that form stable dispersion in both hydrophobic and hydrophilic solvents without any additives. The simple and easy dispersion of **PBN** in a wide range of solvents up to single-layer level represents a significant advance in dispersible 2D materials (Marco et al., 2017). We suspect that the presence of sub-nanometer holes reduces inter-layer interaction while providing strong interaction with some specific small molecules. DFT calculations were performed to understand the dispersion of **PBN** in water.

All dispersion calculations of **PBN** were performed using the Vienna Ab initio Simulation Package (VASP) code (Kresse and Hafner, 1994; Kresse and Furthmüller, 1996). The Perdew–Burke–Ernzerhof (PBE) generalized gradient approximation (GGA) functional and the projected augmented wave (PAW) potential were used to describe the exchange–correlation potential and the ion-electron interactions, respectively. (Perdew et al., 1996; Kresse and Joubert, 1999). The kinetic energy cutoff for the plane-wave base was set to 500 eV and a 2 × 2 × 1 Gamma k-point mesh was used to sample **PBN** and H_2_O molecules. In the case of a fixed unit cell volume, the convergence criterion of 10^−4^ eV for electron energy and convergence criterion of 10^−2^ eV/Å for the forces on each ion were used to optimize all structures. The Many-body dispersion energy method (MBD@rsSCS) was used to determine the interaction energy of PBN and water molecules to account for van der Waals interactions (Lebègue et al., 2010).

In the optimization process of **PBN** and H_2_O structure, we chose gaussian smearing to decide how to set the partial occupancies for each orbital. For the optimized structure, tetrahedron method with Blchl corrections was selected for accurate energy calculation, and the structures of **PBN** and H_2_O were extracted from it, and the same method was used for energy calculation. In order to describe the interaction energy between PBN and water, we define the adsorption energy $\left(E_{\mathrm{ads}}\right)$ as:

$$E_{\mathrm{ads}}=E_{\mathrm{PBN}+H_{2}O}-\;E_{\mathrm{PBN}}-\;E_{H_{2}O}$$

where $E_{\mathrm{PBN}+H_{2}O}$ represents the total energy of **PBN** and water, and $E_{\mathrm{PBN}}$ and $E_{H_{2}O}$ represent the energy of **PBN** and water respectively.

**FIGURE 5:**
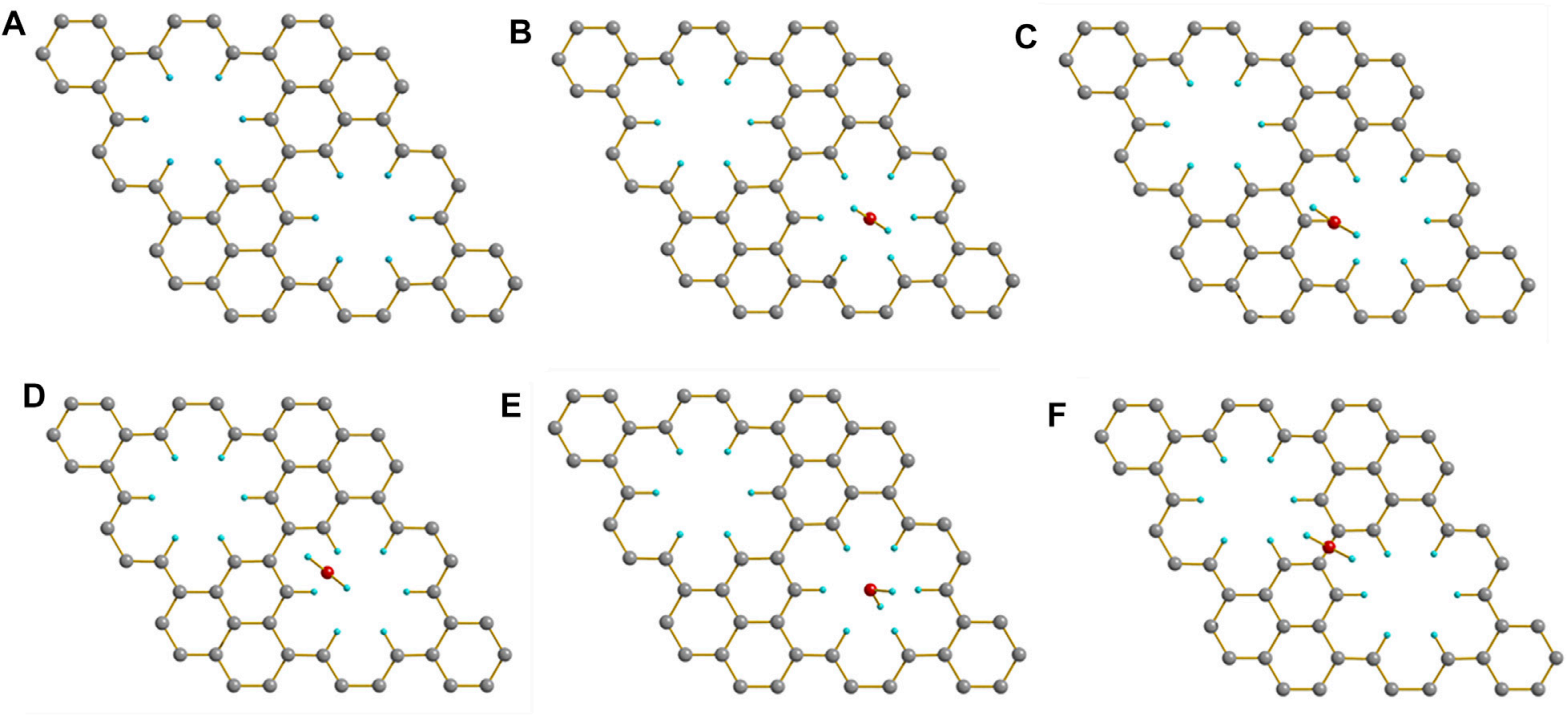
**(A)** The crystal structure of **PBN**. When the water molecule was initially located, **(B)** above the center of the hole, **(C)** above the H atom of the hole, **(D)** above the center of the H-H bond of the hole, **(E)** above the center of the hole, and **(F)** above the entire **PBN**, the optimized crystal structure. Geometric structures were shown through Diamond software.

When **PBN** adsorbed a water molecule, the initial structure and the adsorption energy of the water molecule at different positions is shown in Figure 5. The Figures 5B–E are similar, indicating that the water molecules above the hole will automatically gather to the center of the hole during the optimization process. The holes may have a certain effect on the accumulation of water molecules. In comparison, the adsorption energy was only 0.06 eV when the adsorption site was nearby the center of hexabenzocoronene units, which was actually not unexpected given the bad solubility of large CH aromatics in water. Therefore, the presence of CH decorated holes is crucial to the strong interaction of **PBN** with water molecules.

**FIGURE 6:**
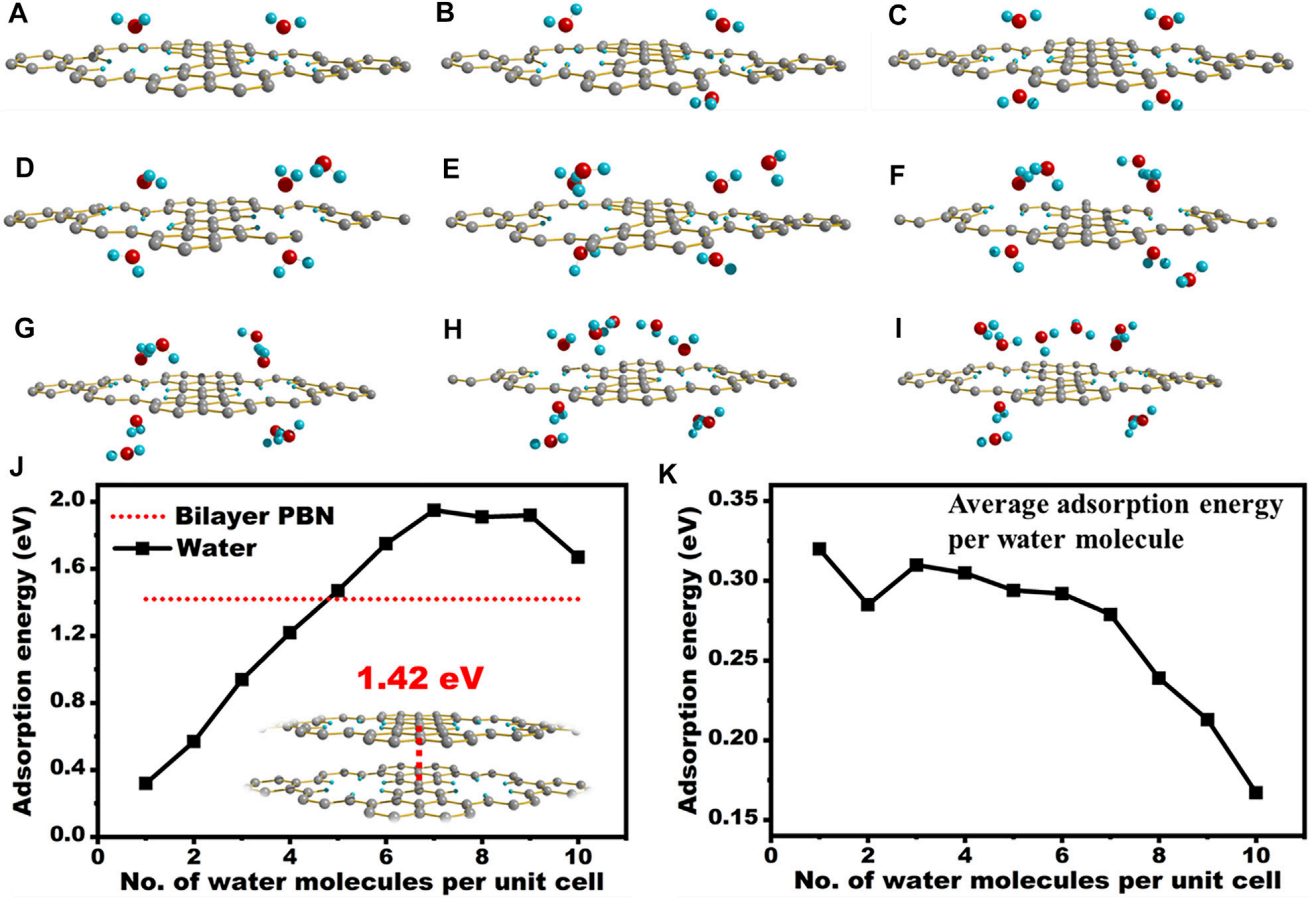
Calculation of the adsorption energy of water interacted with **PBN**. **(A–I)** The optimized structure of **PBN** and two water to ten water molecules, **(J)** The optimized structure of double-layer **PBN** and the absolute value of adsorption energy of different structures, **(K)** The absolute value of adsorption energy per water molecule when **PBN** interacts with different water molecules. Geometric structures were shown through Diamond software.

The adsorption energy of **PBN** with different positions of water is shown in Table 2. The optimized results and the adsorption energy of the first four cases in Table 2 are similar, and they are all greater than the adsorption energy of the last case, indicating that when H_2_O is above the pores, **PBN** has a significant adsorption effect on water. In order to investigate the water solubility of **PBN**, the adsorption energy between **PBN** and multiple water molecules and double-layer **PBN** was calculated, as shown in Figure 6.

**TABLE 2:** The adsorption energy of water at different positions.

Energy	Above the center of the hole	Above the H atom of the hole	Above the center of the H-H bond of the hole	Above the center of the hole	Above the entire PBN
$E_{\mathrm{PBN+}H_{2}O}$ (eV)	−443.78	−443.78	−443.78	−443.79	−443.52
$E_{\mathrm{PBN}}$ (eV)	−429.24	−429.24	−429.24	−429.24	−429.24
$E_{H_{2}O}$ (eV)	−14.22	−14.22	−14.22	−14.22	−14.22
$E_{\mathrm{ads}}$ (eV)	−0.31	−0.32	−0.31	−0.32	−0.06

DFT investigation of the interaction of **PBN** layer with water molecules revealed the interesting role of these sub-nanometer holes. The adsorption energy of one water molecule nearby the holes was around 0.32 eV or 7.34 kcal/mol, which was comparable to or even stronger than common hydrogen bond. In fact, the hydrogen bonding between two water molecules was estimated to be around 0.2 eV. This strong interaction will withdraw water molecule to the vicinity of PBN from the bulk solution. We further increased the number of water molecules per unit cell to probe maximum interaction per hole with water. With the increased in the number of water molecules around the **PBN** pores, the adsorption energy of **PBN** gradually increased, but the increasing trend gradually became flat. When it increased to about five waters, the adsorption energy of **PBN** and water molecules was greater than the interaction energy of double-layer **PBN** molecules. As shown in Figure 6J, as more water molecules were place around the hole, the overall adsorption energy increased almost linearly to 1.9 eV. Excess water molecules may decrease the average adsorption energy per water molecule due to the formation of clusters (Figure 6K). We also computed the interlayer interaction of **PBN**, which was around 1.4 eV per unit cell, and it was actually smaller than the interaction between **PBN** and five water molecules. This explained the good dispersion of **PBN** in water.

## Application of Polyhexabenzocoronene Network in Catalysis

**FIGURE 7:**
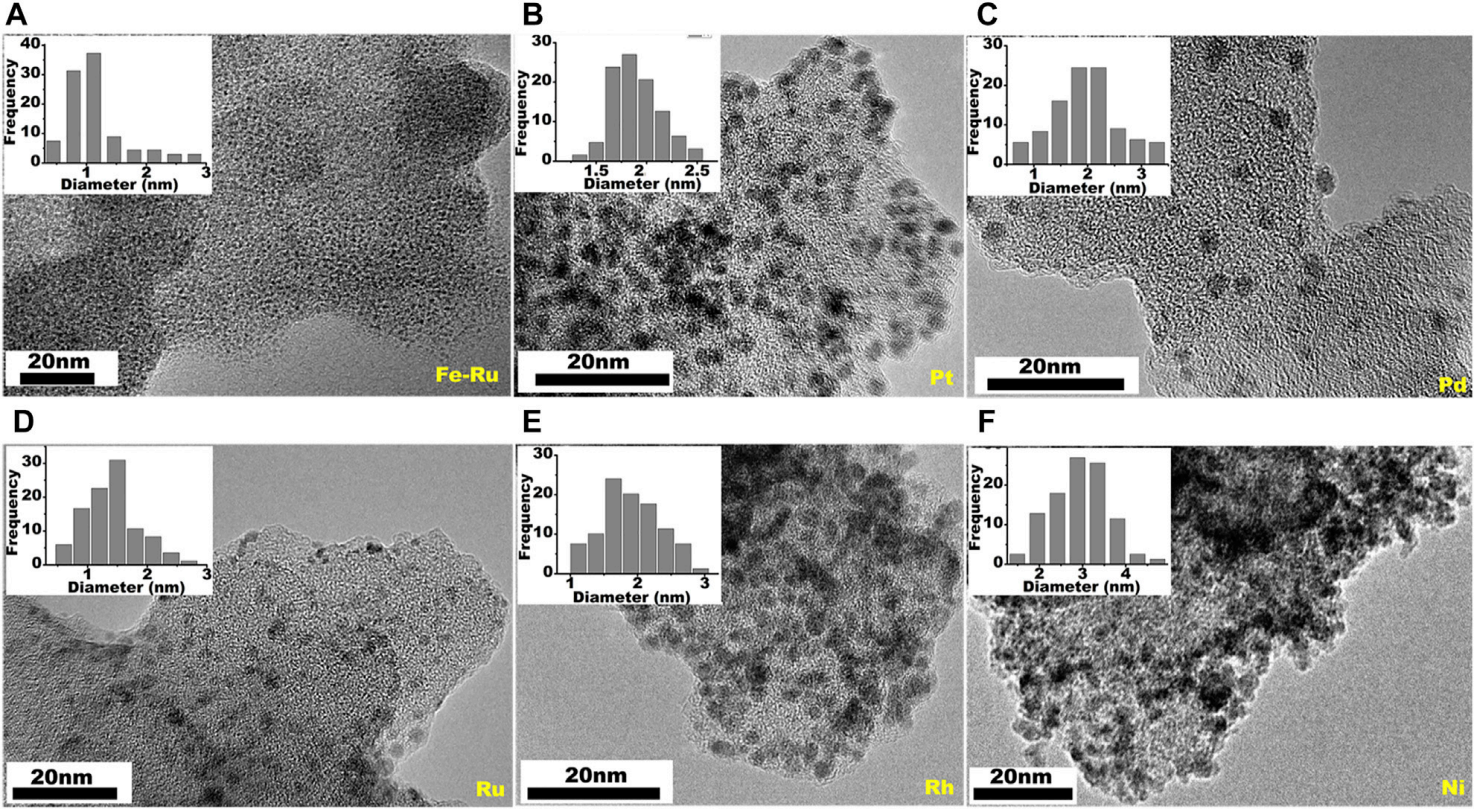
TEM images of **PBN** supported transition nanoparticles of **(A)** Fe-Ru, **(B)** Pt, **(C)** Pd, **(D)** Ru, **(E)** Rh, **(F)** Ni.

Treatment of unwashed **PBN**, which is loaded with FeCl_2_, with excessive NaBH_4_ in ethanol leads **PBN** supported Fe NPs. The low atomic number of Fe prevents direct imaging, but galvanic replace of Fe^2+^ by Ru^3+^ leads to clear view of the extremely small nano Fe particles with sizes around 1 nm (Figure 7A) (Hudson et al., 2015), expected size value from the quantitative cyclodehydrogenation of polyhexaphenylbenzene network (**PHN)**. We further take advantage of the feasible dispersion of **PBN** to combine with various metal precursors in solution in order to expand the scope of loaded NPs. Interestingly, various **PBN-**NPs can be easily prepared simply by chemical reduction of relative metal salts in ethanol in the presence of **PBN**. This simple procedure leads to preparation of supported Pt, Pd, Ru, Rh, and Ni NPs by **PBN** (Figure 7). The sizes of the NPs range from 2 to 5 nm, reflecting the effective confinement of the growth of inorganic NPs by **PBN**. The wide range of supported NPs is consistent with previous DFT calculation which predicates strong interaction between small graphene defects and different various clusters (Navalon et al., 2016).

**FIGURE 8:**
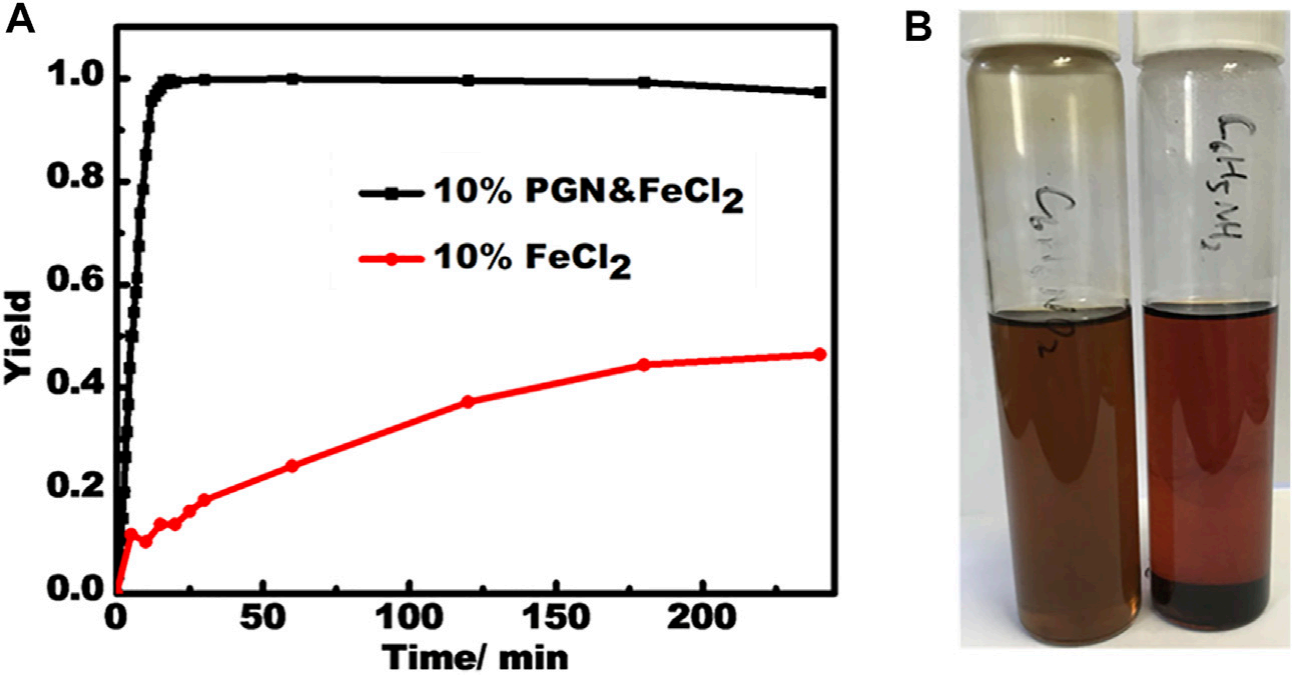
**(A)** The reaction kinetics for catalysts of **PBN**-FeCl_2_ and FeCl_2_. **(B)** The dispersion solutions of **PBN** in nitrobenzene and aniline respectively after 2 h.

The use of these semiconducting **PBN** supported transition metal NPs was preliminarily tested on the reduction of nitrobenzene by NaBH_4_ by use of **PBN**-FeCl_2_ at room temperature. **PBN**-FeCl_2_ exhibits a remarkably different catalysis behavior compared to pure FeCl_2_, which show sluggish first-order reaction kinetics (Figure 8A) (MacNair et al., 2014). **PBN**-FeCl_2_ showed both greatly improved reaction yield and speed at the same 10 wt% loading. The formation of aniline product reaches quantitative within 20 min and the reaction speed remains constant up to around 95% yield, a phenomenon belonging to the zero-order reaction kinetics.

The super performance of **PBN**-FeCl_2_ may be attributed to several factors associated with unique properties of **PBN** mentioned above. First, the zero-order kinetics indicates that there is a strong substrate binding prior to the occurrence of the catalysis on the surface of the Fe NPs, very similar to the enzyme-substrate binding in enzyme catalysis (Koerner et al., 1959). In fact, the **PBN** dispersion in nitrobenzene is stable up to months without significant settlement while **PBN** almost completely settles down in phenylaniline within a few hours (Figure 8B). This dispersion phenomenon indicates the strong binding of reactant and weak binding of the product, a prerequisite for efficient enzyme-like catalysis. Second, together with the small sizes of Fe NPs, their naked nature without any extra functional groups offers more reactive sites to interact with reactants, and therefore speeding up the reaction. Third, the semiconducting nature of **PBN** and its close contact with the Fe nanoparticles may change the electronic nature on the surface through the Mott-Schottky hetero junctions (Su et al., 2017). In addition, filtration test was conducted to validate the nature of heterogeneous catalysis by **PBN**-FeCl_2_.

In addition to its promise in next-generation graphene-based heterogenous catalysts, **PBN** is also attractable for application in gas-filtration (Jiang et al., 2009), water-desalination (Cohen-Tanugi and Grossman, 2012), semiconductor devices (Kim et al., 2011), electromechanical sensors et al. (Boland Conor et al., 2016). where sub-nanometer holes, band-gap or feasible dispersion is required, indicating a wide application space.

## Conclusion

In conclusion, a holey carbon material with ordered sub-nanometer hole defects (polyhexabenzocoronene network, **PBN**) was synthesized by oxidative cyclodehydrogenation with polyhexaphenylbenzene as precursor. The IGM analyses of **PBN** and graphene show that **PBN** has weak interlayer interaction energy compared with graphene, which makes **PBN** has better dispersion performance than graphene. The DFT calculations show that the holes in **PBN** will attract water molecules nearby. With the number of water molecules increased, the interaction energy between **PBN** and water gradually increased, and when it reached saturation, the interaction energy of **PBN** and water was greater than that of double-layer graphene, which confirmed the excellent dispersion of **PBN** in water. It is proved experimentally that **PBN** can be dissolved in many other solvents in addition to water. **PBN** can effectively support various inorganic nanoparticles of small sizes, and when **PBN** supports iron nanoparticles, it has high catalytic activity in the reduction of nitrophenyl by NaBH_4_, indicating that it has great potential in catalysis.

## Data Availability

The original contributions presented in the study are included in the article/Supplementary Material, further inquiries can be directed to the corresponding author.
