# Supplementary material for: Carbon Material With Ordered Sub-Nanometer Hole Defects
Source: Front Chem. 2022 Mar 21;10:858154. doi: 10.3389/fchem.2022.858154 (PMC8979169; doi:10.3389/fchem.2022.858154)
Supplement: Supplementary file 1 [file Table1.DOCX]

Supplementary Material

# Supplementary Tables

**Supplementary Table 1.** Graphic and optimized geometric structure information of **PBN**.

| 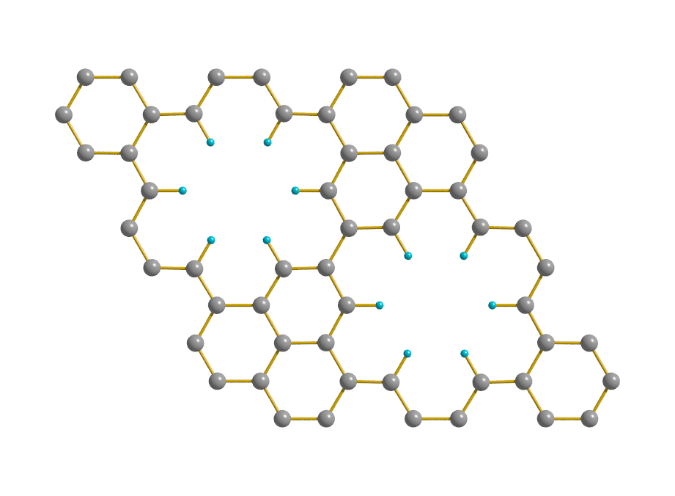 |
| --- |
| 1.00000000000000  12.9184773488941893 0.0000000000000000 -0.0000000000000000  -6.4592386744470947 11.1877295624216195 0.0000000000000000  -0.0000000000000000 0.0000000000000000 29.8340616810911037  C H  42 12  Direct  0.4390182966775124 0.1065909898304065 0.8333299640000007  0.3322228119313408 0.1095570851144495 0.8333299640000007  0.8934089861695915 0.3324273168471067 0.8333299640000007  0.8904429278855480 0.2226657348168848 0.8333299640000007  0.6675726751528928 0.5609817573224849 0.8333299640000007  0.7773342701831122 0.6677772000686604 0.8333299640000007  0.5609817143224848 0.8934090311695917 0.8333299640000007  0.6677772090686647 0.8904429568855469 0.8333299640000007  0.1065909948304034 0.6675726831528935 0.8333299640000007  0.1095570821144528 0.7773342861831135 0.8333299640000007  0.3324272898471081 0.4390182846775114 0.8333299640000007  0.2226657218168872 0.3322228209313381 0.8333299640000007  0.8934090081695935 0.5609817573224849 0.8333299640000007  0.8904429298855481 0.6677772000686604 0.8333299640000007  0.6675726561528947 0.1065909898304065 0.8333299640000007  0.7773342731831160 0.1095570851144495 0.8333299640000007  0.4390183116775171 0.3324273168471067 0.8333299640000007  0.3322228069313369 0.2226657348168848 0.8333299640000007  0.1065909758304054 0.4390182846775114 0.8333299640000007  0.1095570891144499 0.3322228209313381 0.8333299640000007  0.3324273168471067 0.8934090311695917 0.8333299640000007  0.2226657298168878 0.8904429568855469 0.8333299640000007  0.5609817063224842 0.6675726831528935 0.8333299640000007  0.6677772030686642 0.7773342861831135 0.8333299640000007  0.2213555012469027 0.0000000000000000 0.8333299640000007  0.4424013002741288 0.0000000000000000 0.8333299640000007  0.8899319415682980 0.0000000000000000 0.8333299640000007  0.0000000049999968 0.2213555112469035 0.8333299640000007  0.0000000100000009 0.4424013202741234 0.8333299640000007  0.0000000349999993 0.8899320115682966 0.8333299640000007  0.7786444947530972 0.7786445097530948 0.8333299640000007  0.5575987107258756 0.5575987217258730 0.8333299640000007  0.1100680854317008 0.1100680834317007 0.8333299640000007  0.7786444797530995 0.0000000000000000 0.8333299640000007  0.5575986997258713 0.0000000000000000 0.8333299640000007  0.1100680864317044 0.0000000000000000 0.8333299640000007  0.0000000149999977 0.7786445097530948 0.8333299640000007  0.0000000109999973 0.5575987217258730 0.8333299640000007  0.9999999990000035 0.1100680834317007 0.8333299640000007  0.2213555062469066 0.2213555112469035 0.8333299640000007  0.4424013102741297 0.4424013202741234 0.8333299640000007  0.8899319765682973 0.8899320115682966 0.8333299640000007  0.5229201300986055 0.1907491706749199 0.8333299640000007  0.8092507813250760 0.3321709864236773 0.8333299640000007  0.6678289995763217 0.4770798859013958 0.8333299640000007  0.4770798809013990 0.8092508503250782 0.8333299640000007  0.1907491736749237 0.6678290345763210 0.8333299640000007  0.3321710034236822 0.5229201560986005 0.8333299640000007  0.8092508123250750 0.4770798859013958 0.8333299640000007  0.6678290035763220 0.1907491706749199 0.8333299640000007  0.5229201310986020 0.3321709864236773 0.8333299640000007  0.1907491716749235 0.5229201560986005 0.8333299640000007  0.3321710074236826 0.8092508503250782 0.8333299640000007  0.4770798789013988 0.6678290345763210 0.8333299640000007 |
